# Assessment of Potentially Toxic Elements as Non-Point Sources of Contamination in the Upper Crocodile Catchment Area, North-West Province, South Africa

**DOI:** 10.3390/ijerph15040576

**Published:** 2018-03-23

**Authors:** Samuel Che Nde, Manny Mathuthu

**Affiliations:** 1 Department of Geography and Environmental Science, North-West University, Mmabatho 2735, South Africa; 2 Centre for Applied Radiation Science and Technology, North-West University, Mafikeng Campus, Mmabatho 2735, South Africa; Manny.Mathuthu@nwu.ac.za

**Keywords:** upper Crocodile River, contamination, river, sediment, risk assessment, land use

## Abstract

The concentration of potential toxic elements (PTEs) in the Upper Crocodile river catchment area in North-west Province, South Africa, was investigated. Water and sediment samples were collected among different land uses in the upper Crocodile River catchment area and analysed using inductively-coupled plasma–mass spectrometry (ICP–MS). Several guidelines were used to gauge the level of contamination and possible toxic effect of PTEs. The physicochemical analysis showed that electrical conductivity (EC), pH, and total dissolved solids (TDS) values complied with the recommended values of Department of Water and Forestry (DWAF) guidelines for South Africa. The average concentration of Cu, Pb, Cd, Zn, As, Cr, Al, and Mn in the water samples were lower than the recommended levels for water-quality guidelines for aquatic environments except for Fe, which exceeded the recommended values of DWAF of 0.1 mg/L and EPA (US) of 0.3 mg/L. The level of contamination was measured using the enrichment factor, contamination factor, and geoaccumulation index. The level of Cr was above the stipulated threshold limit of the sediment quality guideline for adverse biological effects, suggesting an ecotoxicology risk of anthropogenic origin, which was confirmed by statistical analysis. The non-point sources of PTEs are spatially distributed according to land-use types and are strongly correlated to land use.

## 1. Introduction

The quest for rapid economic growth through industrialisation has had a negative impact on receiving river systems [1]. Anthropogenic activities like agriculture, mining and urbanization have been equally shown to have a negative impact in flowing rivers resulting in degradable aquatic ecosystems and habitats [2]. Different studies have demonstrated that mining, industrial processing and agro-chemicals such as fertilizers and metal-based pesticides, which are toxic to human health, are the main sources of potential toxic element (PTE) concentrations in the environment [3,4,5].

The presence these contaminants (copper (Cu), lead (Pb), cadmium (Cd), zinc (Zn), arsenic (As), chromium (Cr), aluminium (Al), manganese (Mn) and iron (Fe)) in water typically compromises the quality traits expected to be good for drinking, industrial processing and for biodiversity purposes. There is no doubt that South Africa, being a semi-arid country, is faced with a shortage of freshwater resources [6]. Apart from naturally occurring metals in the environments, anthropogenic activities have equally accelerated the rate of toxic metal concentrations in freshwater over the last decades. This situation is being exacerbated by the continuous discharge of wastewater effluent from industrial plants, surface erosion from agricultural areas, mining and waste dumps from the population into nearby river bodies [7,8]. Surface contamination of sediments can act as a sink and secondary source of contamination [9], since they can easily be adsorbed into sediment particles, transported as suspended sediment load, organic matter and nutrients [10], thereby decreasing water quality [11,12], increasing turbidity, restricting light penetration and reducing primary production [13].

Against this background of the national water crisis in South Africa, the Crocodile River in the North-west province, being a semi-arid region (Rustenburg), is no different from the other provinces with water-quality issues. The situation is being exacerbated by many industrial facilities, mining, agriculture as well as a dense population centre [14] which is located not too far from the Crocodile River. Due to decades of intense mining and agriculture, this has had an unprecedented effect causing rapid deterioration of the quality of the river system. A report by the South African Department of Water Affairs and Forestry (DWAF) [15] suggested that surface water in the Crocodile River catchment is being used extensively for agriculture, industry, mining and urban use. Agriculture activities are among the most important, second to platinum group mining in the area. According to van der Walt [16], Impala Platinum produces more than 1.74 million ounces of platinum annually. Due to its rich mineral resources, there is an increased pressure on the natural environment in terms of large-scale industrialisation [14]. However, a perusal of literature in the catchment suggest that baseline quantitative analysis of PTE contamination and sources have not been systematically reported to guarantee the health and safety of the population living in those areas. 

**Figure 1:**
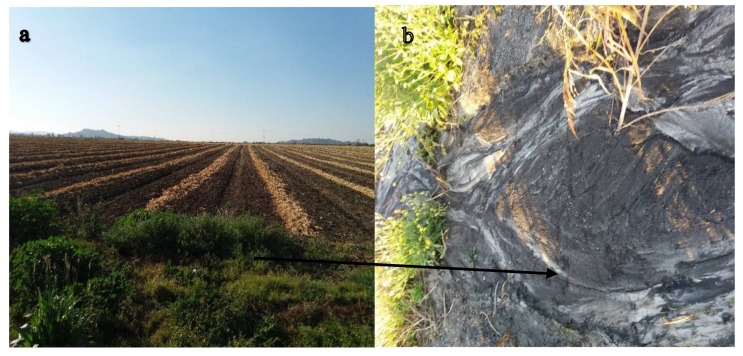
An example of an eroding agricultural field approximately 10 m from the Crocodile River. (**a**) Farrows ready for cultivation; and (**b**) evidence of rill erosion from that cultivated field draining into the Crocodile River, as indicated by the arrow.

Irrespective of their sources in the soil and water, the accumulation of PTEs can degrade soil and water quality [17] when their toxicity levels are above the recommended threshold. According to Ongley et al. [18], there are two types of non-point sources of contamination of particular concern for water quality: agriculture and urban non-point sources. A study by Miller et al. [19] states that non-point sources of contamination are of particular concern in agricultural areas where sediment nutrients and pesticides may negatively impact water quality, as indicated in Figure 1. Other studies have equally reported elevated levels of toxic elements concentrations in surrounding urban, mining and agricultural environments [20,21,22]. Apart from river and soil contamination, the non-carcinogenic risk from exposure to some elements in local aquatic organisms and the riverine population need to be constantly measured to provide critical information about the environment [22,23,24]. This is because once these toxic elements contaminate freshwater resources, they are likely to get into the food chain [25,26] thereby endangering human life. Hence, adequate monitoring of toxic elements has become an important priority over the last decades [23].

A proper management system should be capable of distinguishing multiple sources of contamination within the catchment in order to make a clear distinction between various contributing areas. Although total metal concentration content in soil is useful to estimate the overall contamination potential, soil mobility does not necessarily depend directly on total metal content [25]. Thus, a practical way is to identify the main sources of contamination from different land uses because a host of factors can impose changes on multiple sources of non-point sources under varying hydrological and land use conditions [25,27]. The determination of PTE levels in sediment and aquatic water is of primary concern for the environment besides the need to quantify their concentrations and spatial variability in soil and water. The objectives of this study are, therefore, to: (1) determine the spatial distribution of PTE concentration in water and soil across different land uses along the Crocodile River; (2) assess the degree of contamination in the catchment using indices of contamination. The results will improve our understanding of the possible risk of toxic metal content in the upper Crocodile River catchment, and this is among the few studies that have been conducted on sediment and water in river systems in the province.

## 2. Materials and Methods 

### 2.1. Study Area

**Figure 2:**
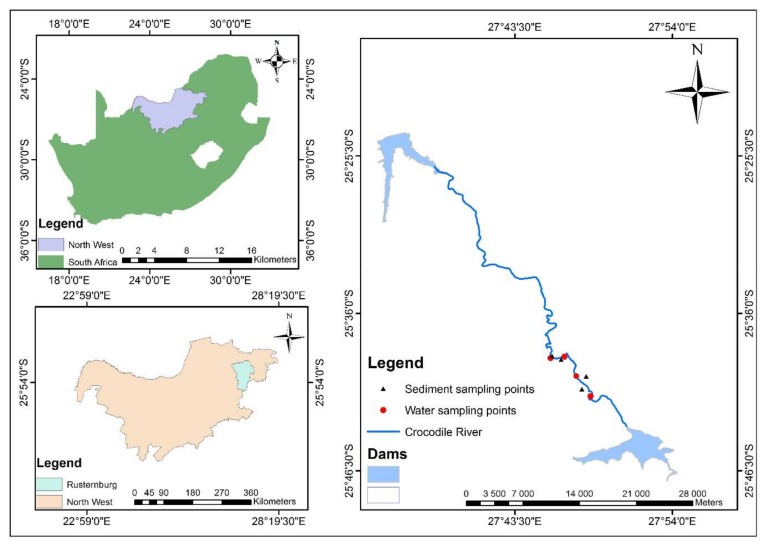
The Crocodile River, showing the sampling points, North-west Province, South Africa.

The upper Crocodile river is one of the sub-catchments in the Crocodile (West) Marico Water Management Area (WMA). The upper Crocodile sub-catchment is situated in the town of Rustenburg which is considered as the economic hub of North-west Province (latitude: −25°40′3.22″ S and longitude: 27°14′31.49″ E, (Figure 2). The town has a population of approximately 549,575 according to Stats SA [28], of which 70% live in the urban area and the remaining 30% in rural areas. The Crocodile River catchment is approximately 41,112 km^2^. It flows in a northward direction and the river traverses a wide variety of lithologies which include shale, dolomite, quartzite, and granophyres [29]. The overbank region of the Crocodile River is under intensive agricultural use, where most irrigation occurs, especially immediately downstream of Hartbeespoort Dam. The area has a number of tourist accommodation resorts along the Crocodile river with sparsely distributed settlements. 

### 2.2. Sediment and Water Collection 

A total of 24 composite soil samples (~500 g), that is 6 per land use, were randomly collected at various distances from an agricultural area, urban area, resort/commercial areas and agriculture/urban area to a depth of 2 cm. These soil samples were stored in zip-lock polystyrene plastic bags and transported to the physical geography laboratory of our institution. Three liters of polyethylene bottles were pre-washed with HNO_3_ and used to collect surface water samples for chemical analysis at different points along the Crocodile River (namely A, B C and D). The sampling strategy ensured that each of the sampling points was within the different land uses (mining, agriculture, commercial and urbanization) and land uses which overlay each other, as presented in Table 1, as areas of non-point sources of contamination. The water samples were collected once every three months in order to take into account changes in seasons (April–September 2017). 

The samples were labeled, and transported to the laboratory for analysis; in situ analysis of surface water were performed upon collection and the following physicochemical parameters were measured at each sampling point: pH, temperature (0 °C), electrical conductivity (EC), total dissolved solids (TDS) using a multimeter (CRISON MM40+). The meter probe was rinsed with distilled water and immersed in the collected water sample for about one minute to reach equilibrium. When the readings of each parameter were constant, the measurements were then recorded on the field data sheet. The same procedure was followed for each of the collected water samples per sampling point.

#### Analytical Methods 

In the laboratory, the soil samples were gently disaggregated using a mortar and pestle and passed through a <2 mm sieve for analysis. Aqua-regia digestion using the Microwave Multiwave 3000, Anton Paar Digestor was performed on a 0.5 g aliquot of the sample using an acid mixture consisting of 69% nitric acid, 70% hypochlorite acid, and 30% hydrogen peroxide, as described by Somerset et al. [23] and Kumar et al. [25]. Water samples were filtered through a filter paper (number 42) into a container to remove all organic impurities and by adding concentrated nitric acid, whereby 10 mL of nitric acid was added to 50 mL of water samples [30]. The following elements were determined using inductive coupled plasma–mass spectrometry (ICP–MS); copper (Cu), lead (Pb), cadmium (Cd), zinc (Zn), arsenic (As), chromium (Cr), aluminium (Al), manganese (Mn) and iron (Fe) in the sediment and water samples. 

### 2.3. Statistical Analysis

The Pearson correlation matrix was used to test for significant relationships between the PTEs in the different land uses in order to identify their sources and occurrence of contamination. Data were processed using Stata (version 13) software. The statistical methods were performed at 95% confidence interval (*p* ≤ 0.05). 

#### PTE Indices in Soil Samples

To estimate the level of contamination in the soil samples in the different land uses, the enrichment factor (EF), contamination factor (CF) and geoaccumulation (*I_geo_*) index were calculated. Their application is based on the principles of comparing the measured values of the elements to the relative amount of the upper continental crust (UCC) of Wedepohl [31]. 

EF has been widely used in different studies as an effective tool to evaluate the magnitude of contamination in the environment [32,33], and to determine if the metals are mostly from lithogenic or anthropogenic origin [34]. Iron (Fe) was chosen as the controlling element in this studies due to its worldwide application and as a reference concentration in uncontaminated areas [33].

(1) $$\mathrm{EF}=\frac{{\left(M/Fe\right)}_{sample}}{{\left(M/Fe\right)}_{background}}$$

where, (*M/Fe*)*_sample_* is the ratio of Fe in the sample of interest and (*M/Fe*)*_background_* is the natural background value of metal to Fe ratio. EF is based on the following categories: ≤1 indicates no enrichment; 1–3 indicate minor; 3–5 indicate moderate; 5–10 is considered moderately severe; 10–25 is severe; 25–50 is considered very severe; and >40 is extremely high enrichment [33].

The contamination factor (CF) is derived from the ratio obtained by dividing the concentration of each metal in the sediment by the baseline or background value [32].

(2) $$\mathrm{CF}=\frac{metal\;contamination}{background\;contamination}$$

Given that need, the following were used to assess the level of contamination factor: CF < 1 (low contamination); 1 ≤ CF < 3 (moderate contamination); 3 ≤ CF < 6 (high contamination factor); CF ≥ 6 (considered very high contamination).

The next step was to quantify the extent of PTE contamination associated with the different land uses by using the geoaccumulation index introduced by Müller [35], and this was defined by the following equation:

(3) $$I_{geo}=Log_{2}\frac{{\left[Sc\right]}_{sediment}}{{\left[Sc\right]}_{background\left(1.5\right)}}$$

where [*Sc*]*_sediment_* is the measured concentration of a given PTE in the sediment sample of in the land use and [*Sc*]*_background_* is the natural background value of the element in the sample. The factor 1.5 is used for a possible variation in the background values due to anthropogenic influences. The geoaccumulation index (*I_geo_*) is interpreted as follows; where *I_geo_* ≤ 0, uncontaminated; 0 ≤ *I_geo_* ≤ 1, uncontaminated to moderately contaminated; 1 ≤ *I_geo_* ≤ 2, moderately contaminated; 2 ≤ *I_geo_* ≤ 3, moderately to highly contaminated; 3 ≤ *I_geo_* ≤ 4, highly contaminated; 4 ≤ *I_geo_*≤ 5, highly to extremely contaminated; *I_geo_* > 5, extremely contaminated [36].

## 3. Results and Discussion

### 3.1. Physicochemical Parameters

Results on the physicochemical parameters at each sampling point indicated fluctuating patterns of the parameters measured, as shown in Table 1. The water temperature at each sampling points indicated a distinct difference between the two seasons, with a minimum of 21 ± 0.1 °C and a maximum of 28 ± 0.2 °C (summer) and 18 ± 0.5 °C to a maximum of 19 ± 0.5 °C (winter) at the time of collection. Although temperature has no direct effect on this study, however, it influences the chemical reaction in water bodies such as corrosion, taste, and the odours of water gases according to DWAF, [15,37]. 

Electrical conductivity is an important parameter in measuring water quality as it gives an indication of the amount of dissolved salts in water. The EC values at each sampling points indicated that the values ranged from a minimum of 511 ± 17 µs/cm to a maximum of 541 ± 12.5 µs/cm, for the wet season, while the dry season had a minimum of 501 ± 7 µs/cm, to a maximum of 523 ± 12 µs/cm. The results were compared with the standards stipulated by DWAF, [15,37], and the values show that all the water sampling points were below the permissive values of less than or equal to (≤400–900 µs/cm) indicating very good to good water quality. 

Results obtained from measuring the pH values at each sampling point during the dry (winter) and wet (summer) season, showed a slight difference from the values observed in both seasons from the different sampling points. Although it has no direct bearing on the water quality, the WHO [38] recommends pH values between 7.0 and 8.5 for drinking water. During the wet season, the maximum values for the pH recorded at each point were 7.5 ± 0.1 while those in dry season recorded a maximum of 8.11, which is below the recommended standard. Although the pH values were generally lower, however, they indicate that the water was slightly alkaline at most points thereby in agreement with the findings of Somerset et al. [23]. 

TDS is another parameter of water quality as it gives an indication of the degree of salinity in the water. It is normally associated with excessive use of fertilizers in agricultural activities or industrial waste discharge into a river [23]. The stipulated guideline by the DWAF [15] recommends a permissive value of 0–450 mg/L for good water quality and 450–1000 mg/L for very good water quality. Results from all the sampling points measured in both wet and dry seasons were <450 mg/L, indicating that the water quality was good. 

### 3.2. Trace Metal Concentration in the Crocodile River 

Table 2 provides the average concentrations of PTEs at different sampling points along the Crocodile River. Most of the elements were within the DWAF [15,37] stipulated guidelines for aquatic environments except for Fe, which exceeded the recommended values of DWAF of 0.1 mg/L and that for EPA (US) of 0.3 mg/L. 

This guideline was exceeded in Point B (agriculture) and D (resort and commercial). Although Fe is an essential element for some living organisms and humans, high levels in water for domestic purposes such as drinking and washing is usually associated with an unpleasant metallic taste and when consumed in a large amount might pose a health risk such as hemochromatosis, a genetic disorder [39]. Cd and As analysis showed that both the elements were far below the detection limit. Cd, on the other hand, is considered toxic to marine and freshwater aquatic life, and its analysis suggests that the element in the river was far below the detection limit suggesting that the river is less toxic. Although Cr in point A (urban) and D (agriculture/mining) were equally below the detection limit, however, point B (agriculture) and C (resort/commercial) values were below DWAF guidelines for aquatic marine environments. 

Generally, results of the dissolved-metal concentrations indicate that the values from the entire sampling point are generally low but this does not exclude the fact that these values might eventually change in future due to enrichment. These changes might be as a result of spatial and temporal input from the environment such as surface runoff from different land uses, catchment sensitivity, settlement dumpsites, agriculture, and dilution due to precipitation [39]. For instance, a study by Odiyo et al. [40] indicated emission from automobiles and waste disposal as major sources of heavy-metal concentrations to the Mvudi Rivers around Thohoyandou. Therefore, there is a need to constantly gauge the level of these metals’ toxicity and to keep the riverine population informed of the potential health risk associated with farming activities and fishing when operating within this vicinity.

Further analysis was used to compare the result of this study to previously reported studies (Table 3). 

First, the researcher chose a study in the same catchment management area and one in another province. An analysis of the results showed contrasting values between the case study and the present study. The average concentration of Cu was significantly greater than those reported by Sumerset et al. [23] and Edokpyi et al. [39]. Edokpyi et al. [39] found out that Pb was below the detection limit in the Mvudi River, whereas, the values in this study exceeded those reported by Sumerset et al. [23]. This, therefore, signifies a slight increase in the concentration of Pb in the Crocodile River. Similarly, the average concentration of Cd was below the detection limit in this study, but there was a slight difference between the two reported studies by Edokpyi et al. [39] and Sumerset et al. [23]. Zn, As, Cr, Al, Mn and Fe were not reported in the study by Sumerset et al. [23] but their analysis in this study showed a significant difference between the average concentration of Cr, Al, Mn and Fe in the Mvudi River and in the Crocodile River, except for As which was below the detection limit but was not available in the Mvudi River. These results, however, should be interpreted with caution due to the interplay of other factors operating in the area such as land use, rainfall, and catchment sensitivity. 

### 3.3. Potential Toxic Element (PTE) Concentration in Sediment Samples

The results of metal concentrations in sediment samples indicate that average concentrations of the elements in the sediments varied according to land use and follow the order; Fe > Al > Mn > Cr > Zn > Cu > Pb > As >Cd (Table 4). 

Although Fe and Mn are the most abundant metals in sediment [41], the concentration of Fe is relatively low to Al with high concentrations in resort/commercial areas followed by the agriculture/mining area, then by the urban and agricultural area. The extent of metal concentrations (Al, Cr, Mn Zn and Fe) observed in the agriculture/mining area were found to be higher than those reported by OLowoyo et al. [42] conducted in a mining area in South Africa. Hu et al. [27], reported that the concentration of PTEs varied spatially according to urban, agricultural and industrial land use. 

To assess the possible ecotoxicological risk of the PTE contamination in the sediment samples, this study used two guideline values proposed by Long et al. [43], that is: the effect range-low (ERL) and effect range-median (ERM) (Table 4). The results indicate that the concentration of Cr in all the land uses exceeded both the ERL and ERM values, indicating a potential ecotoxicology risk. In addition, the values of Cr exceeded 100 mg kg^−1^ for uncontaminated soil proposed by Kabata-Pendias [44]. The high concentration of Cr in the study catchment may pose a serious health threat to the communities living around that area as well as an ecotoxicology effect on vegetal organisms [45]. Cr is also known to affect lungs, causing lung cancer and death [42]. 

#### Estimation of Contamination Intensity in the Different Land Uses

The results from the calculation of enrichment factor (EF), contamination factor (EF) and geoaccumulation index (*I_geo_*) for the PTEs are presented in Table 5. In geochemical studies, EF is used to differentiate between PTEs originating from natural sources with those from anthropogenic sources [33]. According to Diop et al. [34], an EF < 1.5 indicates that the elements are entirely from crustal origin or natural processes while an EF > 1.5 suggests the elements are likely from anthropogenic sources. The results reveal Cu and Cr in all the land uses were >1.5, suggesting anthropogenic origin, whereas As and Mn were >1.5 only in the agriculture and agriculture/mining areas. 

Furthermore, the EF for these PTEs ranges in order of moderately severe to very severe in agricultural land use, urban land use, resort/commercial land use and agriculture/mining land use, respectively, reiterating the close association between contamination of those elements in the various land uses most likely from anthropogenic sources. No enrichment for Cd, Zn and Al in all the land uses suggests that these elements are entirely from crustal materials. Cu indicates minor enrichment in all the land uses; Pb and As display minor enrichment only in agricultural land use (Table 5).

Generally, the CF (see Table 5) values of Cd, Pb, Zn, As and Al indicate that these elements in the different land uses show low contamination while Cu, Mn and Al in the other land uses indicate moderate contamination, except for Cr with moderate contamination in agricultural land use, while urban, resort/commercial and agriculture/mining land uses had high CF, respectively. The *I_geo_* results according to Table 5 reveal that the values of Cu, Pb, Cd, Zn, As, Mn and Al in all the land uses (except Cr in agriculture land use) were less than zero, suggesting that these land uses are not contaminated. The *I_geo_* classes for Cr in urban land use and resort/commercial land use indicate they are moderately contaminated, while the agriculture/mining land use was heavily contaminated according to the classification of Müller [46], suggesting its concentration is heavily affected by anthropogenic inputs and surface runoff from nearby contamination sources [26]. This result, thereby, confirms the results obtained from EF and CF evaluation. 

### 3.4. The Correlation between PTEs in the Sediment Samples

The values of the Pearson correlation coefficients (*r*) of the metal concentrations in the sediment samples and water samples analysed for the different land uses as possible non-points sources are given in Table 6 and Table 7. 

The results indicate that there is a strong positive correlation between metal concentrations in the sediment samples of the different land uses (*r* = 0. 99). 

Analysis of Table 6 indicates that there is a positive significant correlation between urban and agriculture metals (*r* = 0.88), urban and resort/commercial (*r* = 0.94), urban and agriculture/mining (*r* = 0.94), agriculture and resort/commercial (*r* = 0.98), agriculture and agriculture/mining (*r* = 0.94) and resort/commercial and agriculture/mining (*r* = 0.99). The positive correlation of the different land uses suggest that these elements have the same sources and are most likely due to anthropogenic activities, thereby confirming the results of the EF and emanating from the same sources probably influenced by the use of agro-fertilizers, discharges from urban and industrial plants, and waste disposal from privately owned resort accommodations.

In the case of the metals in the water samples, there was a positive correlation found between metal concentration among the different sampling points (*r* = 96) as indicated in Table 7.

The results indicate a positive correlation between urban and resort/commercial (*r* = 0.98), urban and agriculture/mining (*r* = 0.95), resort/commercial and agriculture/mining (*r* = 0.90) indicating that the sources of these metals are most likely influenced by anthropogenic activities of the same source. However, a weaker correlation was observed between urban and agriculture (*r* = 0.45), agriculture and resort/commercial (*r* = 0.50), and agriculture and agriculture/mining (*r* = 0.48), suggesting that their spatial distribution in the river is area specific being influenced by the corresponding land use draining into the Crocodile River, as shown in Figure 1. Although the deterioration in available water quality can be linked to these land uses it should, however, be noted that the processes often occur gradually, and consequently environmental sustainability will suffer in the long run [36]. Thus, adequate measures are needed to reduce water deterioration, especially in those areas which are considered as sources of contamination.

## 4. Conclusions

This study concluded that the Crocodile River is contaminated with toxic elements, as Fe was found to be higher than is recommended by DWAF and USEPA. However, As, Cd, Cr, Cu, Pb and Zn were present in lower concentrations than the stipulated guideline values for aquatic life, but this does not exclude the fact that these values might eventually change in the future if they are considered over a continuous period of time due to the continuous discharge of contaminants into the river from privately owned resorts and accommodation and fertilizers from agricultural sites, as was observed during the field visit. This was confirmed, as the values of Cu and Pb were higher than the previously reported study in the catchment. The results of the metals analysis showed that the EF of Cr in the soil samples ranges in order of moderately to severe enrichment, revealing anthropogenic sources. This was supported by the CF ranging from moderately to high CF and *I_geo_* also indicating moderately to highly contamination. In addition, the concentrations of Cr were above the ERL threshold limit in agriculture and urban land use, and the ERM threshold limit in resort/commercial and agriculture/mining land use of sediment quality guidelines for adverse biological effects, which may constitute an ecological risk as well as a risk to the population. 

The positive correlations exhibited by the soil and water samples from the different land uses indicated that these elements are spatially distributed, having similar behaviour to a common source. Non-point sources of metals in the soil and river could possibly be attributed to anthropogenic activities such as agriculture, mining, resorts and privately owned accommodation, commercial activities and increasing population along the Crocodile River. A measure to curb metal pollution in the Crocodile River would be to avoid tannery discharge effluent into the river and farmland without prior treatment. Apart from the treatment of wastewater effluent discharge into the Crocodile River, it is imperative to adopt alternatives measures of cleaning up already existing contaminated substrates. Periodic monitoring of the soil and water rate of contamination and the consumption of fish from the river is, thus, necessary in order to assess the overall exposure level in the riverine communities, depending on the river.

## Figures and Tables

**Table 1:** Physicochemical parameters from various sampling points.

Water Sampling Points	Site ID	Temp. (°C)		EC (µs/cm)		pH		TDS (mg/L)	
		Wet Season	Dry Season	Wet Season	Dry Season	Wet Season	Dry Season	Wet Season	Dry Season
Point A	Urban	27.3 ± 1.2	19.9 ± 1.3	529 ± 11.3	519 ± 4.3	7.5 ± 0.1	8.2 ± 0.1	332.6 ± 2	380.6 ± 5.5
Point B	Agriculture	28.3 ± 0.2	19.8 ± 1.3	517 ± 7	511 ± 1	7.3 ± 0.2	8.9 ± 0	330.6 ± 3.5	427 ± 0
Point C	Agriculture/Mining	21.1 ± 0.1	19.5 ± 0.5	511 ± 17	523 ± 12	7.3 ± 0.2	8.11 ± 0	330.6 ± 3.5	320 ± 6
Point D	Resort/Commercial	25.3 ± 0.5	18.9 ± 0.5	541 ± 12.5	501 ± 7	7.4 ± 0.2	8.7 ± 0.1	339.3 ± 20.8	321 ± 4.5
DWAF*		N/A*		400–900		5.0–9.5		450–900	
WHO*		N/A*		N/A*		7.0–8.5		N/A*	
EPA*		N/A*		N/A*		6.5 ≥ pH ≤ 8.5		500	

N/A* = Not Available; DWAF* = Department of Water Affairs and Forestry, South Africa; EPA* = (US Environmental Protection Agency); WHO* = World Health Organisation; EC = electrical conductivity; TDS = total dissolved solids.

**Table 2:** Results showing the average heavy-metal concentrations collected at each sampling point in the Crocodile River.

Site ID	Cu	Pb	Cd	Zn	As	Cr	Al	Mn	Fe
A	0.03 ± 0.00	Bdl*	Bdl*	0.05 ± 0.01	Bdl*	Bdl*	0.12 ± 0.06	0.05 ± 0.02	0.07 ± 0.03
B	0.04 ± 0.01	Bdl*	Bdl*	0.04 ± 0.02	Bdl*	Bdl*	0.04 ± 0.03	0.04 ± 0.02	0.28 ± 0.22
C	0.03 ± 0.02	Bdl*	Bdl*	0.04 ± 0.04	Bdl*	0.01 ± 0.00	0.08 ± 0.08	0.06 ± 0	0.06 ± 0.05
D	0.08 ± 0.55	0.02 ± 0.01	Bdl*	0.10 ± 0.54	Bdl*	Bdl*	0.17 ± 0.02	0.05 ± 0.04	0.11 ± 0.03
DWAF*	<2	0.01	00.003	5	0.01	0.05 mg/L	<0.005 mg/L	0.18 mg/L	0.1 mg/L
WHO*	<2	0.01	<0.003	N/A	0.01	0.05 mg/L	N/A	0.40	N/A
EPA*	0.3	0.015	0.005	5	0.01	N/A	N/A	0.05	0.3

DWAF* = Department of Water Affairs and Forestry, South Africa. EPA* = (US-Environmental Protection Agency). WHO* = World Health Organisation. Bdl* = Below detection limit.

**Table 3:** Comparisons of heavy-metal contaminations South African rivers.

Trace Metals	Mvudi River [39]	Crocodile (WMA) [23]	Crocodile River [This Study]
Cu	0.011–0.593	0.002–0.011	0.03 ± 0.00–0.08 ± 0.05
Pb	Bdl–0.046	0.008–0.02	Bdl–0.02 ± 0.01
Cd	0.002–0.0043	0.003–0.009	Bdl*
Zn	0.001–0.548	N/A**	0.04 ± 0.02–0.10 ± 0.45
As	N/A*	N/A**	Bdl*
Cr	0.012–0.548	N/A**	Bdl–0.01 ± 0.0
Al	0.0393–13.81	N/A**	0.12 ± 06–0.17 ± 0.02
Mn	0.029–0.675	N/A**	0.04 ± 0.02–0.61 ± 0.00
Fe	0.0425–5.07	N/A**	0.07 ± 0.03–0.28 ± 0.22

N/A* = not available; Bdl* = below detection limits; N/A** = not reported.

**Table 4:** Metal concentration (mg/kg) in sediment samples from different land uses.

Land Uses						
PTEs	Agriculture	Urban	Resort/Commercial	Agriculture/Mining	ERL	ERM
Cu	18.65 ± 3.40	22.45 ± 2.15	22.83 ± 0.90	23 ± 5.01	34	270
Pb	10.80 ± 4.30	6.24 ± 0.44	6.53 ± 0.44	5.85 ± 0.71	46.7	218
Cd	0.05 ± 0.03	0.05 ± 0.01	0.05 ± 0.0	0.05 ± 0.01	1.2	9.6
Zn	25.64 ± 20.12	22.24 ± 2.03	36.32 ± 6.73	30.06 ± 4.26	150	410
As	1.82 ± 0.93	1.01 ± 0.08	0.9 ± 0.13	0.69 ± 0.40	8.2	70
Cr	99.0 5 ± 20.99 ^a^	262.45 ± 17.17 ^a^	822.95 ± 583.35 ^b^	882.43 ± 187.07 ^b^	81	370
Al	1328 3 ± 5167	38,552 ± 2629	29,440 ± 5905	24,495 ± 4441	N/A	N/A
Mn	821.05 ± 539.9	660.05 ± 39.20	764.60 ± 90.40	716.25 ± 66.03	N/A	N/A
Fe	17,265 ± 5749	20,257 ± 2136	28,555 ± 2773	24,407 ± 5088	N/A	N/A

PTEs = potential toxic elements. ^a^ Concentrations that exceed the ERL (effect range-low). ^b^ Concentrations that exceed ERM (effect range-median).

**Table 5:** Soil contamination assessment for PTEs in the sediment samples collected at various land uses.

PTEs	Agriculture			Urban			Resort/Commercial			Agriculture/Mining		
	EF	CF	*I_geo_*	EF	CF	*I_geo_*	EF	CF	*I_geo_*	EF	CF	*I_geo_*
Cu	2.33	1.3	−0.20	2.39	1.56	0.06	1.72	1.59	0.08	2.03	1.6	0.10
Pb	1.23	0.63	−0.59	0.55	0.36	−2.03	0.41	0.38	−1.96	0.43	0.34	−2.12
Cd	0.74	0.49	−1.61	0.74	0.49	−1.61	0.53	0.49	−1.61	0.62	0.49	−1.61
Zn	0.88	0.49	−1.60	0.65	0.42	−1.81	0.75	0.69	−1.10	0.73	0.57	−1.37
As	1.6	0.41	−0.72	0.77	0.5	−1.57	0.04	0.45	−1.73	0.43	0.34	−2.12
Cr	5.06	2.83	0.91	11.43	7.49	2.32	25.43	23.51	3.97	31.9	25.21	4.07
Al	0.3	0.17	−3.12	0.75	0.49	−1.59	0.41	0.38	−1.98	0.4	0.31	−2.24
Mn	2.78	1.55	0.05	1.9	1.25	−0.2	1.56	1.45	−0.04	1	1.35	−0.14

**Table 6:** Pearson correlation coefficients (*r*) of PTEs in the soil samples from different land uses in the upper Crocodile River catchment.

Land Uses	Urban	Agriculture	Resort/Commercial	Agriculture/Mining
Urban	-			
Agriculture	0.88 *	-	-	
Resort/Commercial	0.94 *	0.98 *	-	-
Agriculture/Mining	0.94 *	0.98 *	−0.99 *	-

Pearson correlation metric of the soil samples across the different land uses. * represents significance at *p* ≤ 0.05.

**Table 7:** Pearson correlation coefficients (r) of PTEs in the water samples from different land uses in the upper Crocodile River catchment.

Land Uses	Site ID	Urban	Agriculture	Resort/Commercial	Agriculture/Mining
Urban	A	-			
Agriculture	B	0.45	-	-	
Resort/Commercial	C	0.95 *	0.50	-	-
Agriculture/Mining	D	0.95 *	0.48	−0.90 *	-

Pearson correlation metric of the water samples from different land uses. * represents significance at *p* ≤ 0.05.
